# Screening and identification of potential novel biomarker for diagnosis of complicated *Plasmodium vivax* malaria

**DOI:** 10.1186/s12967-018-1646-9

**Published:** 2018-10-04

**Authors:** Hargobinder Kaur, Rakesh Sehgal, Archit Kumar, Alka Sehgal, Devendra Bansal, Ali A. Sultan

**Affiliations:** 1 Department of Medical Parasitology, Postgraduate Institute of Medical Education and Research, Chandigarh, 160012 India; 2 Department of Virology, Postgraduate Institute of Medical Education and Research, Chandigarh, India; 3 Department of Obstt. & Gynae, Government Medical College and Hospital, Chandigarh, India; 4 Department of Microbiology and Immunology, Weill Cornell Medicine-Qatar, Cornell University, Qatar Foundation-Education City, Doha, Qatar

**Keywords:** *Plasmodium vivax*, Complicated *P. vivax* malaria, miRNA, Biomarker

## Abstract

**Background:**

In the recent years *Plasmodium vivax* has been reported to cause severe infections associated with mortality. Clinical evaluation has limited accuracy for the early identification of the patients progressing towards the fatal condition. Researchers have tried to identify the serum and the plasma-based indicators of the severe malaria. Discovery of MicroRNA (miRNA) has opened up an era of identification of early biomarkers for various infectious and non-infectious diseases. MicroRNAs (miRNA) are the small non-coding RNA molecules of length 19–24 nts and are responsible for the regulation of the majority of human gene expressions at post transcriptional level.

**Methods:**

We identified the differentially expressed miRNAs by microarray and validated the selected miRNAs by qRT-PCR. We assessed the diagnostic potential of these up-regulated miRNAs for complicated *P. vivax* malaria. Futher, the bioinformtic analysis was performed to construct protein–protein and mRNA–miRNA networks to identify highly regulated miRNA.

**Results:**

In the present study, utility of miRNA as potential biomarker of complicated *P. vivax* malaria was explored. A total of 276 miRNAs were found to be differentially expressed by miRNA microarray and out of which 5 miRNAs (hsa-miR-7977, hsa-miR-28-3p, hsa-miR-378-5p, hsa-miR-194-5p and hsa-miR-3667-5p) were found to be significantly up-regulated in complicated *P. vivax* malaria patients using qRT-PCR. The diagnostic potential of these 5 miRNAs were found to be significant with sensitivity and specificity of 60–71% and 69–81% respectively and area under curve (AUC) of 0.7 (p < 0.05). Moreover, in silico analysis of the common targets of up-regulated miRNAs revealed UBA52 and hsa-miR-7977 as majorly regulated hubs in the PPI and mRNA–miRNA networks, suggesting their putative role in complicated *P. vivax* malaria.

**Conclusion:**

miR-7977 might act as a potential biomarker for differentiating complicated *P. vivax* malaria from uncomplicated type. The elevated levels of miR-7977 may have a role to play in the disease pathology through UBA52 or TGF-beta signalling pathway.

**Electronic supplementary material:**

The online version of this article (10.1186/s12967-018-1646-9) contains supplementary material, which is available to authorized users.

## Background

Malaria is a one of the most important life-threatening parasitic infection affecting human beings. Out of the five species of Plasmodium genus responsible for causing infection in humans, *Plasmodium falciparum* and *Plasmodium vivax* are the two most important responsible for causing the majority of the malaria infections [1]. Globally 4% of the malaria infections are caused by *P. vivax* alone and India is one of five countries responsible for causing 85% of *P.vivax* infections in 2016 [2]. Over the past few years, the belief of *P. vivax* responsible for causing benign infections in humans is being challenged by the reports of morbidity and mortality caused by the severe *P. vivax* infections [3–6]. There have been increasing reports of the presentation of severe *P. vivax* malaria which includes the symptoms ranging from altered sensorium, seizures, cerebral malaria, jaundice, acute respiratory distress syndrome (ARDS) shock, acute kidney injury (AKI) and severe anaemia [7–11].

In the recent years with the implementation of molecular diagnosis, it has become possible to demonstrate *P. vivax* as a solo cause for the underlying multi organ dysfunctions and the life-threatening conditions similar to those caused by *P. falciparum* [12]. However, microscopy is routinely being used for the diagnosis of malaria and remains the gold standard. Also rapid antigen detection test (RDT) and molecular tests (PCR and LAMP) have also been used for the diagnosis of malaria [13]. But we do not have any technique that can be used in order to differentiate the complicated *P. vivax* malaria from the uncomplicated *P. vivax* malaria. Therefore, early diagnosis of these complicated *P. vivax* and accurate treatment is an important means to prevent the progression of uncomplicated *P. vivax* malaria to a severe form and ultimately death.

An ideal biomarker for a disease should be less-invasive, stable and present in body fluids in enough amounts for easy diagnosis of the disease [14]. miRNA could represent itself as an ideal marker for being stable, single stranded, small, non-coding, RNA molecule which is evolutionary conserved and has an important role in regulating the translation of mRNA. miRNAs are 19–24 nts long, regulating majority of the human gene expressions at the post transcriptional level by targeted RNA degradation and translational arrest [15]. The researchers have established a significant correlation between the miRNA and the cause of the underlying disease. Recently, the potential role of miRNA as biomarker has been widely studied in many infectious and non-infectious diseases [16–19]. In various viral, bacterial and parasitic infections the dysregulation of these miRNA has been elucidated and well correlated with the underlying disease pathologies [20]. Among parasitic diseases, in schistosome infection, the potential role of circulating miR-223 as new biomarker and the assessment of the response to chemotherapy have been reported [21]. Tiwari et al. [22] have reported that consistent dysregulation of 85 miRNA in Leishmania infection with the specific involvement of 10 miRNAs in the regulation of macrophage effector functions in infected macrophages. Among the Apicomplexan parasites, in *T. gondii*-infected mice, plasma miRNAs were found to be differentially expressed suggesting the potential role as an early biomarker of *T. gondii* infection [23]. The role of miRNAs in host response to *Cryptosporidium* has also been well elucidated in number of functional studies [24]. However, a few studies have examined the role of miRNA in malaria specifically. In malaria the pathophysiological role of miRNA has not been well established [25]. Although, there are few studies explaining the probable role of these miRNA as a putative biomarker for *P. vivax* malaria but none explains the potential for their use as a biomarker for severe *P. vivax* malaria. Here, in the present study, the differential miRNA expression in whole blood samples of complicated *P. vivax* group of patients were studied using miRNA microarray and the potential of miRNA was then explored to be used as an early predictor of complications in *P. vivax* malaria.

## Methods

### Sample classification for miRNA microarray and qRT-PCR

In total, 48 whole blood samples have been collected for miRNA studies. The samples have been classified into four groups as shown in Table 1. Group I included healthy controls which are negative for *P. vivax* by multiplex nested PCR. Group II and III patients were positive for *P. vivax* by microscopy, confirmed by molecular techniques (multiplex nested PCR, Real time PCR and LAMP) [26, 27]. The samples in these groups were classified as complicated and uncomplicated *P. vivax* on the basis of the WHO based criteria for severe malaria patients [28]. Group IV consisted of *P. falciparum* positive patients, which were confirmed by microscopy and multiplex nested PCR. Group I and Group IV subjects were enrolled for miRNA microarray only. The demographic and clinical details from the enrolled patients were obtained at the time of sample collection.

**Table 1:** Classification of patient and control samples into four groups (Group I-IV)

	Group I	Group II	Group III	Group IV
Criteria	Healthy individuals	Patients infected with *P. vivax* (complicated malaria)	Patients infected with *P. vivax* (uncomplicated malaria)	Patients infected with *P. falciparum*
Number of samples (For microarray analysis)	4	6	4	2
Number of samples (For q RT PCR)		16	16	
Total samples	4	22	20	2

### Sample collection

Approximately, 4 ml of blood sample was taken from all subjects by a trained practitioner. For microscopy and molecular detection methods ~ 1.0 ml of the sample was collected in EDTA vacutainer. And 2.5 ml of the sample was collected in the PAXgene blood RNA tubes (PreAnalytics-BD, New Jersey, USA) and kept at − 20 °C for 24 h and later stored at − 80 °C for further use.

### RNA extraction

On the day of RNA isolation, the samples were allowed to thaw completely at room temperature (18–25 °C) for at least 2–3 h. From the whole blood samples, the total RNA was extracted using the PAXgene blood RNA kit (Qiagen, Germany) as per the manufacturer’s instruction. The concentration and purity of the total RNA was estimated using NanoDrop-1000 Spectrophotometer (Thermo Fisher Scientific Inc., Wilmington, DE) by measuring 260/280 ratio. The RNA with ratio of 1.9–2.2 were further processed for real time PCRs. The integrity of the respective samples was also checked by doing the native gel electrophoresis.

### miRNA microarray using Affymetrix miRNA 4.0 array (format 100 midi)

For miRNA microarray, the total RNA was isolated as mentioned above following manufacturer’s instructions. The RNA integrity number was assessed using Agilent’s Bioanalyzer 2100 (Agilent Technologies Inc., USA) for each sample. As per the affymetrix miRNA protocol of miRNA 4.0 array; the steps of fragmentation and hybridization was performed followed by the scanning using Affymetrix scanner 3000 7G. The miRNA 4.0 array provided a comprehensive coverage for miRNAs for human (Affymetrix, Santa Clara, CA, USA). The microarray raw data was extracted from the files (raw intensity file) generated during scanning of slides. These raw data sets were separately analyzed using Expression Console and Trancriptome console software followed by a differential miRNA expression, fold change and cluster analysis.

#### Endogenous control selection for normalization of qRT-PCR data

As per the previous published studies of the endogenous controls (EC) used for whole blood samples, in total five genes (3 snoRNA and 2 miRNA) namely U6, U44, U48, miR425 and miR16 were selected to test their potential as an endogenous control in our study [16, 29, 30]. In total of 18 samples (10 complicated and 8 uncomplicated) were used to identify the suitable endogenous controls for qRT-PCR.

#### Candidate EC stability analysis

geNorm, NormFinder, bestkeeper alogrithms and ΔCt ± SD methods were used to assess the stability of the candidate ECs. geNorm was used to rank the endogenous controls as per their stability values (M) representing the variation in expression of each EC in comparison to each other [31]. Normfinder is a Microsoft excel add-in tool that take in account both inter and intra group variation (complicated and uncomplicated). This gives the stability value; the lower the value the more stable will be the EC gene. The converted quantities (2^−ΔCt^ (ΔCt = the corresponding Cq value-minimum Cq) [31] were used for analysis in both geNorm and Normfinder analysis.

#### Candidate miRNA selection criteria for validation by quantitative Real time PCR (qRT-PCR)

**Fig. 1:**
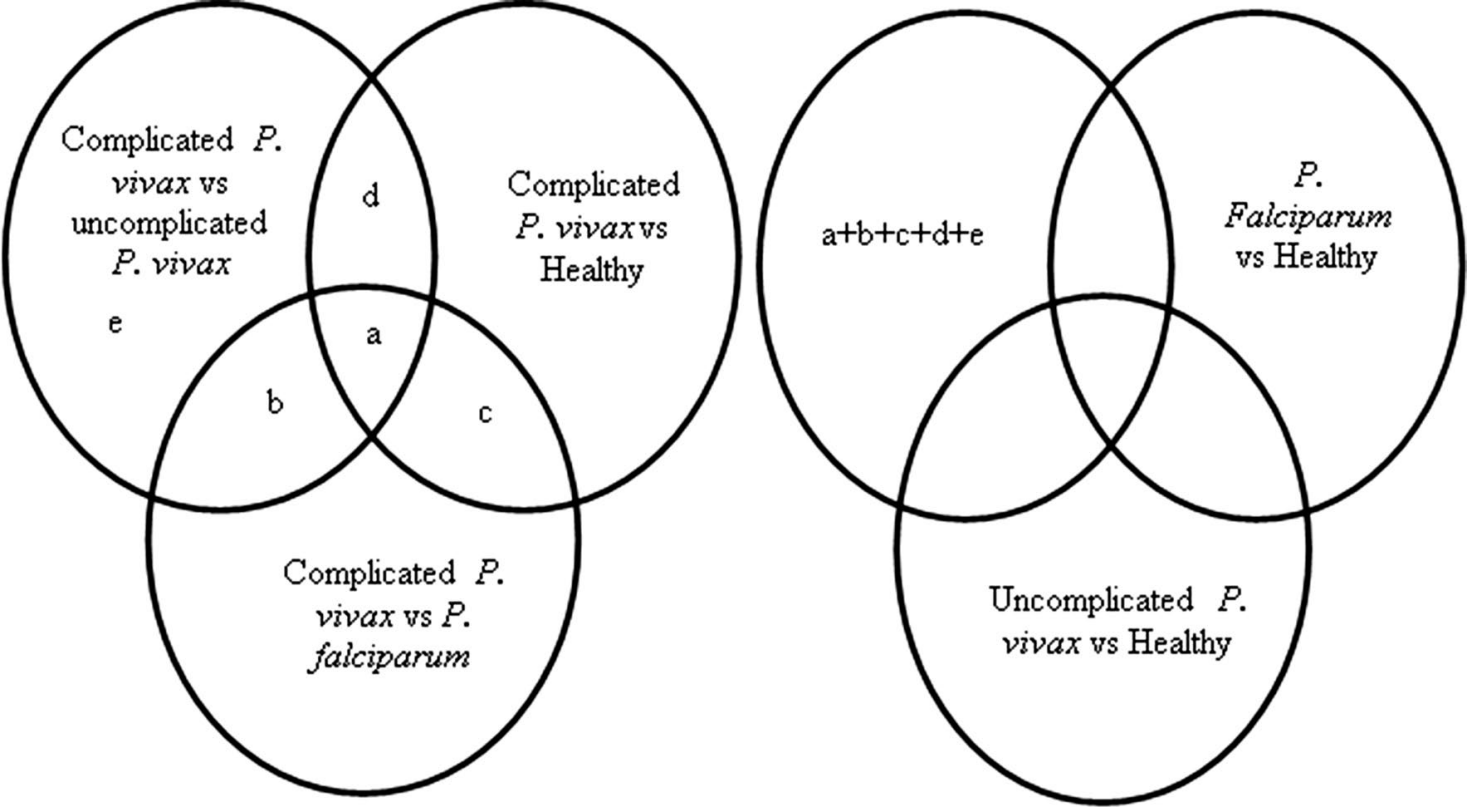
Venn diagram analysis showing the overlap of the up-regulated miRNAs

A profile of differentially expressed small non-coding RNA was obtained from miRNA microarray with 100% miRBase v20 coverage. For validation by qRT-PCR, the selection of mature miRNAs was made by making venn between the complicated vs uncomplicated *P. vivax*, complicated *P. vivax* vs healthy and complicated *P. vivax* vs *P. falciparum.* The miRNAs which were then found to be common in any of the two or three groups with a fold change of FC ≥ 3.5 and p < 0.05 and miRNAs in complicated vs uncomplicated *P. vivax* group with a fold change of FC ≥ 5.0 and p < 0.05 were then selected. Out of the selected miRNA, those miRNAs were removed from further analysis which were found to be common in *P. falciparum* vs healthy and uncomplicated *P. vivax* vs healthy groups as shown in Fig. 1.

A total of 12 mature miRNAs were selected for validation by qRT-PCR. The primer sequences used for these 12 mature miRNAs are shown in Table 2. Specific miRNA forward and universal reverse primers were used for qRT-PCR.

**Table 2:** Mature miRNA primer sequence used for q RT PCR

S. No	Gene	Forward primer (5′-3′)
1	hsa-miR-7977	TTCCCAGCCAACGCACCA
2	hsa-miR-28-3p	CACTAGATTGTGAGCTCCTGGA
3	hsa-miR-378a-5p	CTCCTGACTCCAGGTCCTGTGT
4	hsa-miR-194-5p	TGTAACAGCAACTCCATGTGGA
5	hsa-miR-3667-5p	AAAGACCCATTGAGGAGAAGGT
6	hsa-miR-3651	CATAGCCCGGTCGCTGGTACATGA
7	hsa-miR-192-5p	CTGACCTATGAATTGACAGCC
8	hsa-miR-19b-3p	TGTGCAAATCCATGCAAAACTGA
9	hsa-miR-181b-5p	AACATTCATTGCTGTCGGTGGGT
10	hsa-miR-29a-3p	TAGCACCATCTGAAATCGGTTA
11	hsa-miR-200c-3p	TAATACTGCCGGGTAATGATGGA
12	hsa-miR-155-5p	TTAATGCTAATCGTGATAGGGGT

#### miRNA validation: cDNA synthesis and quantitative Real time PCR (qRT-PCR)

For cDNA synthesis, 1 µg of total RNA sample for each isolate was reverse transcribed in a final volume of 20 µl using the miRNA 1st-Strand cDNA Synthesis Kit (Agilent Technologies, Santa Clara, California) as per manufacturer’s instruction. Briefly, polyadenylation step was first performed at 37 °C for 30 min and termination of the reaction was carried out at 95 °C for 5 min. After obtaining the polyadenylated product, the 1st strand cDNA synthesis was performed at 55 °C for 5 min, 25 °C for 15 min, 42 °C for 30 min and finally incubated at 95 °C for 5 min to terminate reverse transcription reaction. The aliquots of the obtained cDNAs was diluted to 15-fold and stored at − 20 °C for further use. The selected 12 miRNAs were quantified using Maxima SYBR Green/ROX qPCR Master Mix (2X) (Thermo Scientific, USA) in a 20 µl reaction, consisting of 1× mastermix, 200 nM of primers (miRNA specific forward and universal reverse) and 2 µl of cDNA. The housekeepings were run simultaneously with each sample reaction. The qRT-PCR was performed in 96 well plate using the Applied Biosystems 7500 step one real-time PCR system (Applied Biosystems, Carlsbad, CA, USA) using a three-step protocol of 40 cycles of 95 °C for 10 s, 58 °C for 15 s and data was acquired at 72 °C for 15 s. Each sample was run in triplicates. ΔΔCt method was used to carry out the relative quantitation for obtaining the results for each miRNA. The ΔCts were calculated as difference of Ct values between the target miRNAs and the housekeeping gene.

#### Diagnostic potential of putative biomarker of complicated *P. vivax* malaria

The ROC curve was generated for the miRNAs, which were significantly up-regulated by qRT-PCR in the complicated *Plasmodium vivax* group. The area under curve (AUC) value and 95% confidence intervals were calculated to estimate the sensitivity and specificity of significantly up-regulated miRNAs. All the statistical analysis was carried out using Graph Pad prism. version 5.01.

#### Target gene selection, functional and pathway enrichment analysis

The qRT-PCR up-regulated miRNAs were then further selected for target gene analysis using TargetScan [32]. The list of potential targeted genes was obtained individually for each miRNA. The DAVID server with default setting was used to analyze the involvement of target genes (of all up-regulated miRNA individually and combined) in biological processes and for functional analysis [33]. The KEGG and reactome pathways were used to exemplify the pathways of the predicted miRNA target genes using functional annotation tool of DAVID using human genes as background set. The cut off of p < 0.01 was used for the identification of potential enriched KEGG pathways.

#### Network construction and analysis

The target genes common between any of the two miRNAs were then further used for the construction of protein- protein interaction network using STRING database [34]. To study the protein–protein interaction the data obtained from STRING was visualized in Cytoscape Software (Cytoscape Software, Version 2.8.2, Seattle, USA) using the group attributed layout based on the degree of all nodes [35]. The mRNA–miRNA network was constructed using nodes with degree of more than 20 in cytoscape and visualized using degree sorted circular layout.

### Statistical analysis

To determine the differential expression of mature miRNAs between the complicated and uncomplicated *P. vivax* groups, Mann–Whitney U test was applied. The *p *< 0.05 was considered as statistical significance. All the statistical analysis was carried out using Graph Pad prism. version 5.01.

## Results

The male to female ratio and median age [interquartile range (IQR)] of the patients in complicated, uncomplicated group was found to be 2.7:1, 20 (11–27) years and 0.8:1, 12 (6–29) years respectively. In the healthy group (n = 4), two males and two females with median age of 16 (4–28) years were enrolled in the study and they were not found to have any clinical symptoms of malaria or any other major illness at the time of sample collection. In *P. falciparum* group, the mean age of the patients was 19 years and they were possessing the acute febrile illness with the presence of severe thrombocytopenia, epistaxis, severe anaemia, jaundice with renal impairment. The 20 uncomplicated *P. vivax* patients were found to have general symptoms of malaria such as chills and rigors associated fever with general body weakness. The 22 complicated *P. vivax* patients recruited in the present study had one or the other complications as defined by the WHO criteria for severe malaria. The major complications presented in the 22 complicated group of patients included jaundice 27% (n = 6), seizures with altered sensorium 27% (n = 6), renal impairment, abnormal bleeding, severe anemia 9% (n = 2 each), and ARDS in one patient.

### Differentially expressed miRNA in complicated *Plasmodium vivax*

**Fig. 2:**
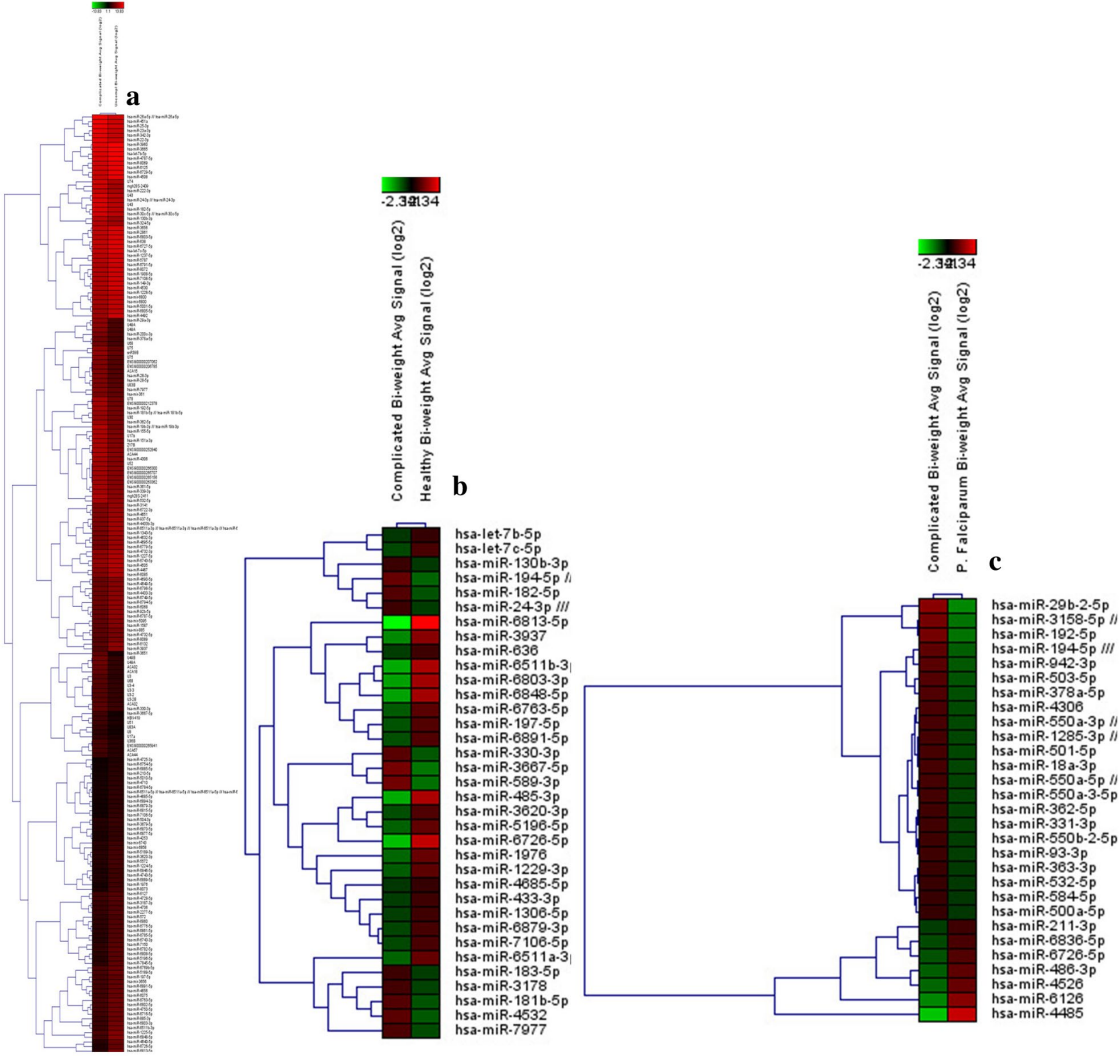
Heat map analysis representing the differential expression of miRNA among different groups. **a** Complicated vs uncomplicated *P. vivax.* **b** Complicated *P. vivax* vs healthy. **c** Complicated *P. vivax* vs *P. falciparum*. Red color shows over-expressed miRNAs (> 0) & green color shows under-expressed miRNAs (< 0). (The heat map image has been generated on the basis of log2 normalized intensity value)

To interrogate all miRNAs in miRBase Release 20, Affymetrix miRNA 4.0 array is designed. On comparing the profile of miRNA obtained in microarray in the groups, complicated vs uncomplicated *P. vivax*, complicated *P. vivax* vs Healthy and complicated *P. vivax* vs *P. falciparum* a total of 276 miRNA were found to be differentially expressed (data shown in Additional file 1: Table S1). Out of 276, 155 matute miRNA (32 up-regulated and 123 downregulated), 35 mature miRNAs (12 up-regulated and 23 downregulated), 30 mature miRNAs (22 up-regulated and 8 downregulated) were found to be differentially expressed in complicated vs uncomplicated *P. vivax*, complicated *P. vivax* vs Healthy and complicated *P. vivax* vs *P. falciparum*. The expression patterns of these miRNAs are displayed in the clustered heat map shown in Fig. 2.

#### Candidate miRNA selection for quantitative real time PCR validation

**Fig. 3:**
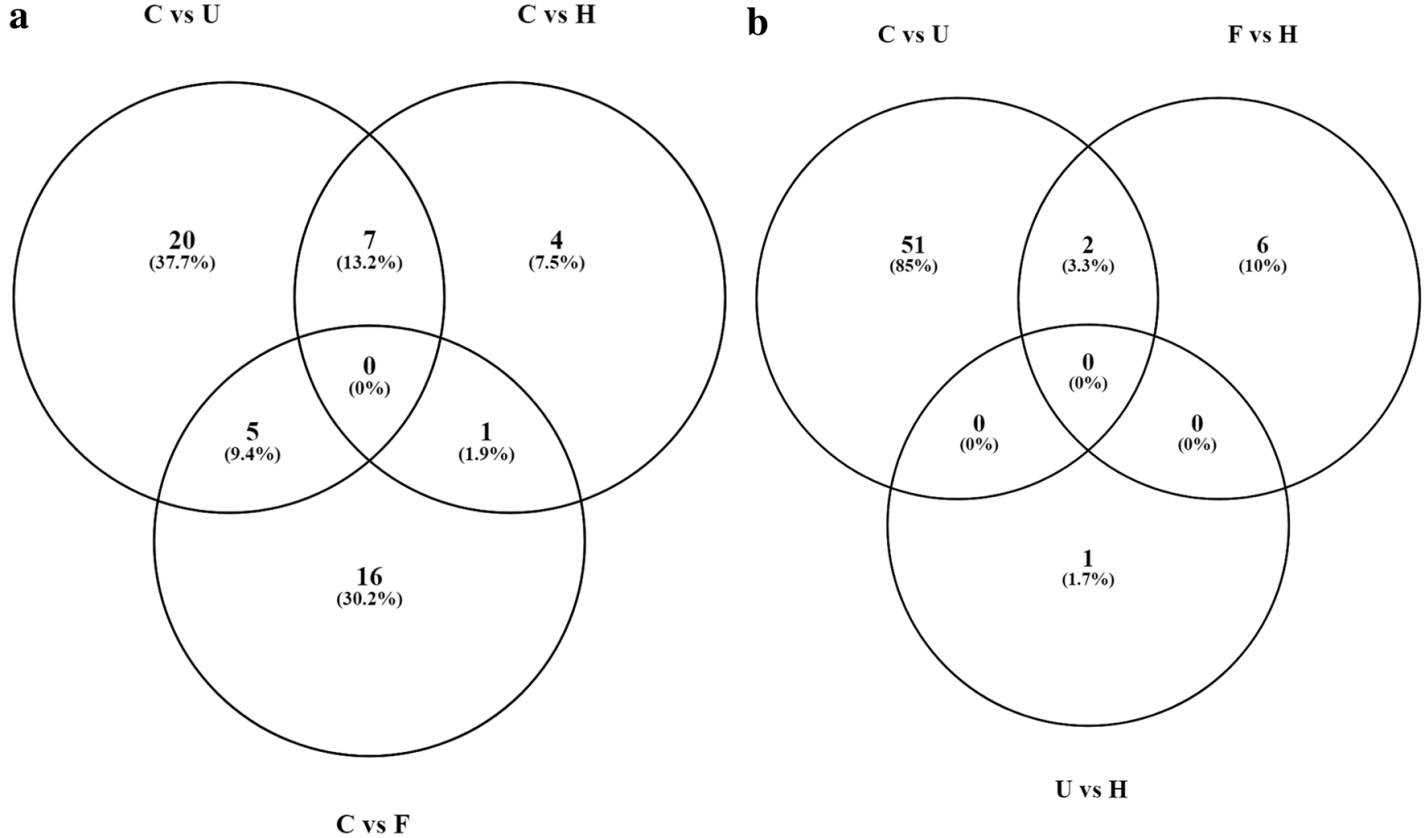
Venn diagram analysis showing the overlap of the up-regulated miRNA between **a** C vs U, C vs H, C vs F **b** C (all miRNA of complicated groups), F vs H, U vs H (C: complicated *Plasmodium vivax*; U: uncomplicated *Plasmodium vivax*; H: healthy; F: *Plasmodium falciparum*) [62]

In total 12 mature miRNAs (hsa-miR-7977, hsa-miR-28-3p, hsa-miR-378a-5p, hsa-miR-194-5p, hsa-miR-3667-5p, hsa-miR-3651, hsa-miR-192-5p, hsa-miR-19b-3p, hsa-miR-181b-5p, hsa-miR-29a-3p, hsa-miR-200c-3p, hsa-miR-155-5p) were selected for qRT-PCR validation. Six with FC ≥ 3.5 and p < 0.05 were selected on the basis of their presence in any of the two groups (Fig. 3) and 6 miRNAs with FC ≥ 5.0 and p < 0.05 in complicated vs uncomplicated *P. vivax* group was chosen for validation in a larger number of samples by qRT-PCR.

#### Selection of candidate housekeeping for quantitative real time PCR (qRT-PCR)

The geNorm, Normfinder, BestKeeper and Ct values ± SD were used to assess the stability of five selected genes (U48, U6, U44, miR-425 and miR-16) (Table 3).

**Table 3:** Candidate reference genes stability as per four statistical algorithms

miRNA	geNorm		Normfinder		Best keeper			Ct values ± SD		Geometric mean (Rank)
	Rank	Stability (M)	Rank	Stability (ρ)	Rank	Std dev [± CP]	r, BKI	Rank	Mean ± SD	
U48	1	1.313	1	0.050	3	2.07	0.871; p = 0.001	3	22.47 ± 2.31	1.7
U6	2	1.464	4	0.80	1	0.83	0.450; P = 0.02	1	24.16 ± 1.06	1.6
U44	4	1.668	5	0.095	2	1.49	0.718; p = 0.001	2	24.64 ± 1.77	2.9
miR-425	3	1.482	2	0.058	4	2.48	0.914; p = 0.001	4	22.87 ± 2.96	3.1
miR-16	5	2.282	3	0.067	5	2.73	0.618; p = 0.001	5	22.44 ± 3.15	4.4

*R* Pearson’s linear correlation coefficient, *BKI*  BestKeeper index, *SD* standard deviation

#### geNorm

**Fig. 4:**
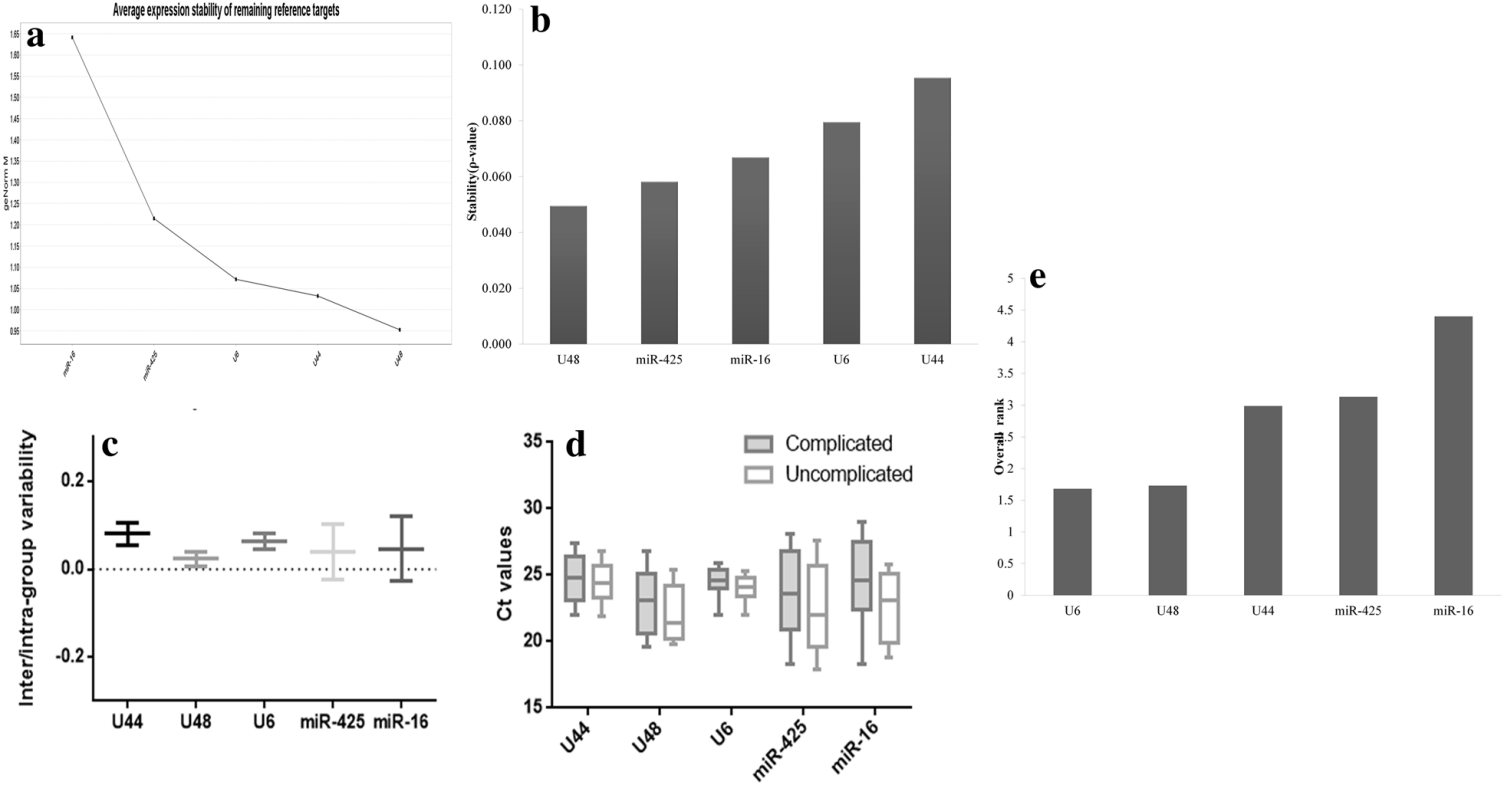
Selection of housekeeping gene out of five candidate housekeeping genes (U6, U44, U48, miRNA-425 and miRNA-16). **a** geNORM analysis showing M value. **b**, **c** NormFinder showing the stability of 5 genes on basis of ρ value and inter and intra group variability between putative genes. **d** Ct values ± SD method showing Ct value and standard deviation of all five genes. **e** Overall geometric mean showing U6 and U48 at position first

In term of stability (M value), U48 and U6 came out to be most stable gene with M value of 1.3 and 1.4 respectively. In geNorm following the procedure of step-wise exclusion of the candidate gene, miR-16 turned out to worst reference gene with the highest M value of 2.2 (Fig. 4a).

#### Normfinder

As per the Normfinder analysis, the U48 was the most stable gene with the stability index (ρ) of 0.050 and was found to have the least values of intra-group variability (0.023). The stability of the two genes U6 and U44 was found to be the worst with high ρ values of 0.80 and 0.095 (Fig. 4b).

#### BestKeeper

In terms of variability, U6 displayed the lowest standard deviation value of 0.83 in Bestkeeper analysis. Correlation analysis was performed and BestKeeper index (BKI) was calculated for the candidate genes. On the basis of BKI, U48 and miR-425 showed the highest correlation of r > 0.87 and r > 0.91 and p = 0.001 and U6 had a lower correlation value (r < 0.45 and p = 0.02) (Fig. 4c).

#### Comparative Ct values ± SD method

Similar to geNorm and BestKeeper, this method also showed miR-16 to be the least stable candidate gene with the highest SD of Ct values of 3.15 respectively. U6 was found to be the most stable gene with the average SD values of 1.06 respectively (Fig. 4d).

On the basis of the calculated geometric mean of ranks, U6 and U48 was found to be at the top rank and thus used for the further quantitative real time PCR experiments (Fig. 4e). miR-16 and miR-425 were found to be on the lowest rank as per combined analysis.

#### qRT-PCR validation

**Fig. 5:**
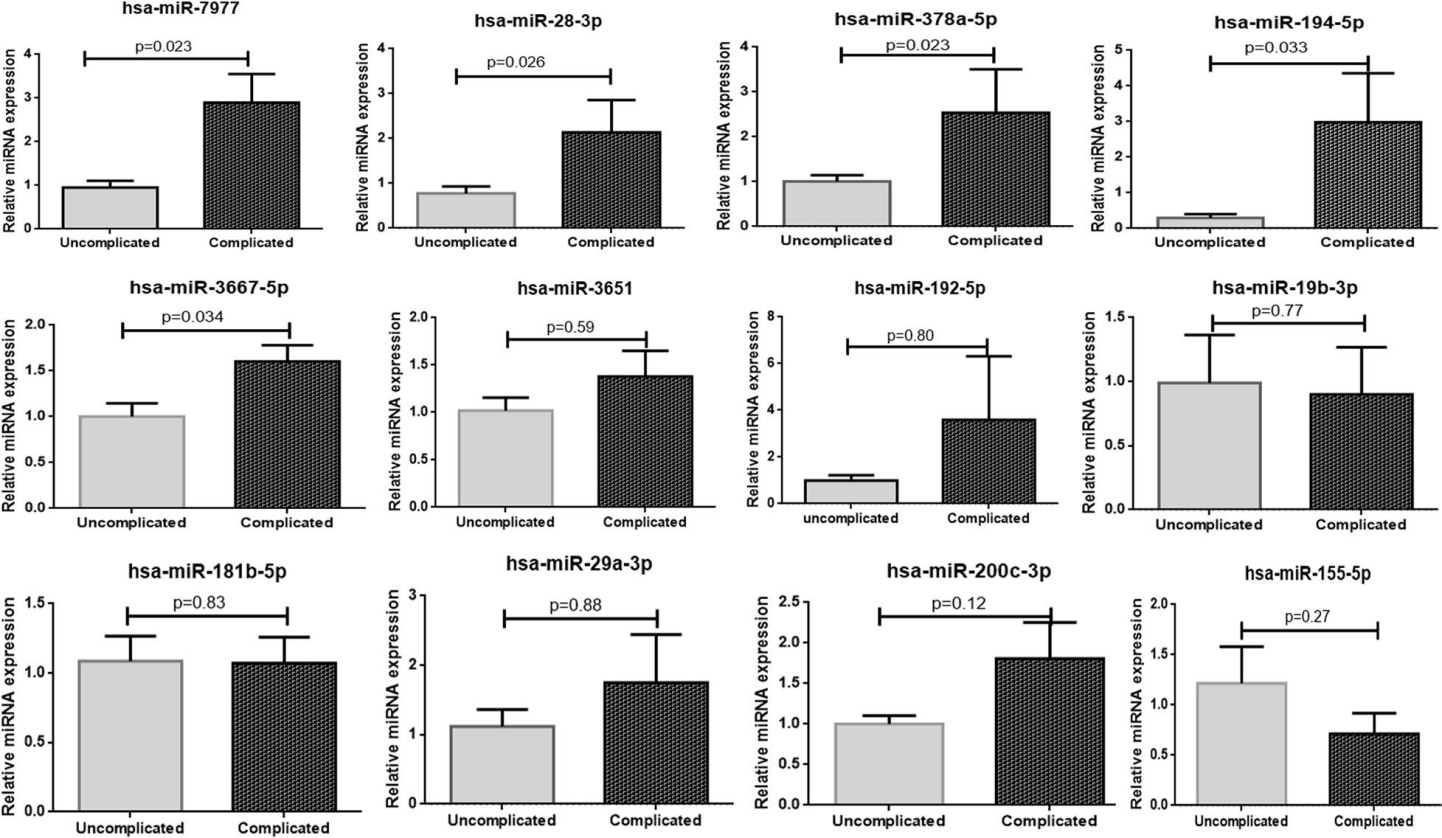
q RT PCR validation of 12 selected miRNA in both complicated and uncomplicated *P. vivax* group of patients (Mann–whitney U test; p < 0.05)

Out of a large number of differentially expressed miRNAs, a total of 12 mature miRNAs were selected (criteria mentioned above) for further validation by using q RT-PCR SYBR green method in both complicated and uncomplicated group of patients. The results of candidate miRNAs were normalized to the geometric mean of two selected housekeeping genes U6 and U48. Out of 12 miRNAs, the expression of 5 miRNAs hsa-miR-7977, hsa-miR-28-3p, hsa-miR-378a-5p, hsa-miR-194-5p, hsa-miR-3667-5p were found to be statistically significantly (p < 0.05) up-regulated in the complicated *P. vivax* group as compared to the uncomplicated *P. vivax* group of patients (Fig. 5). No significant difference in the expression was obtained for the rest of the 7 miRNAs.

#### Evaluation of the diagnostic potential of 5 up-regulated miRNA for complicated *Plasmodium vivax* infections

**Fig. 6:**
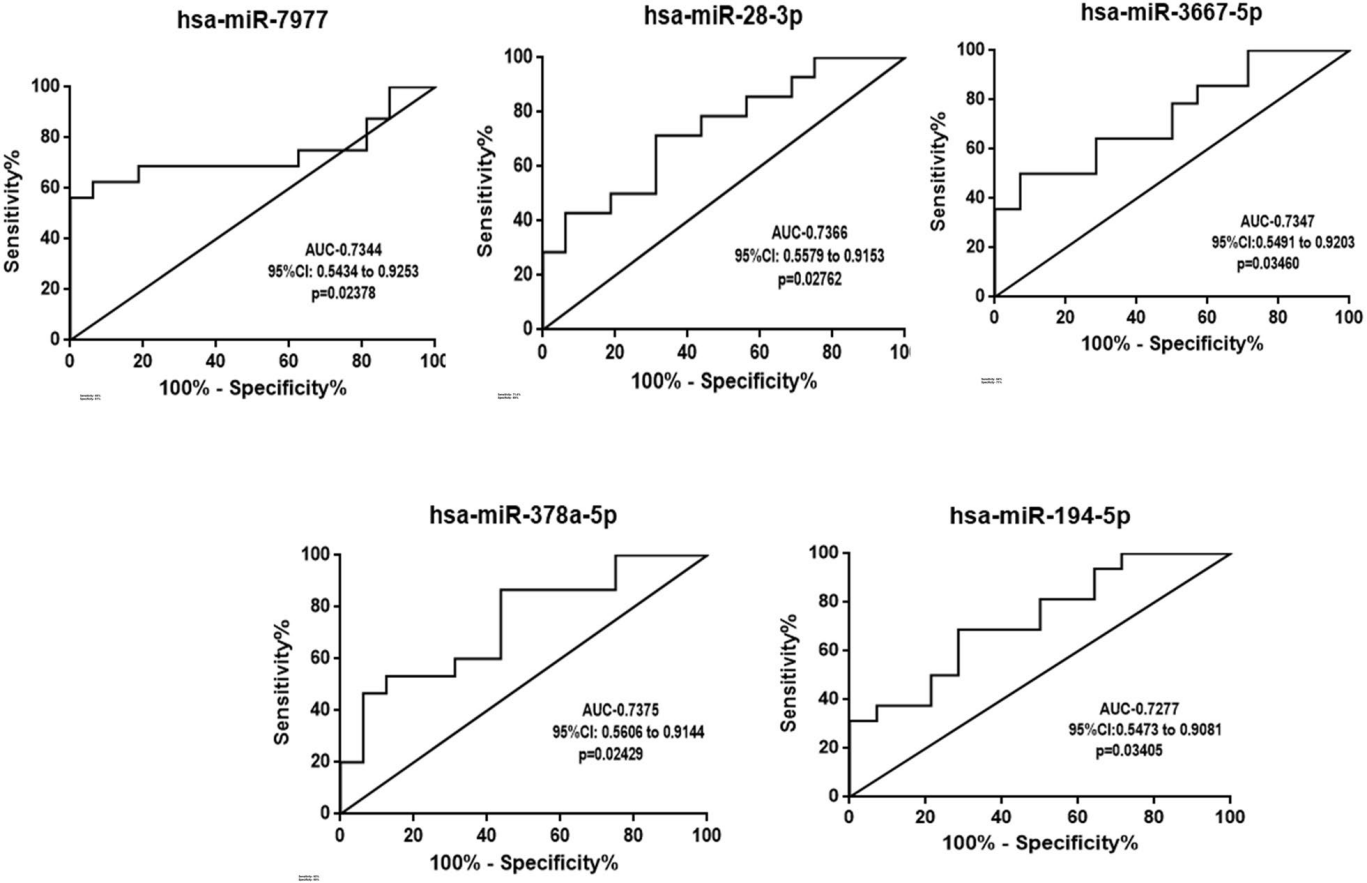
Receiver operating characteristic (ROC) curve analysis for all five up-regulated miRNAs as predictor of complicated *P. vivax* malaria

To investigate the potential of five significantly up-regulated miRNAs (hsa-miR-7977, hsa-miR-28-3p, hsa-miR-378-5p, hsa-miR-194-5p, hsa-miR-3667-5p) to be used as putative biomarker for *P. vivax* complicated infections the Receiver operating characteristic (ROC) curve analysis was performed for all five miRNAs individually. The hsa-miR-7977 represented the area under curve (AUC) of 0.7344; 95% CI 0.5434 to 0.9253; p = 0.02378, hsa-miR-28-3p with AUC = 0.7366; 95% CI 0.5579 to 0.9153; p = 0.02762, hsa-miR-378-5p with AUC = 0.7347; 95% CI 0.5606 to 0.9144; p = 0.02429)], hsa-miR-194-5p with AUC = 0.7277; 95% CI 0.5473 to 0.9081; p = 0.03405 and hsa-miR-3667-5p with AUC = 0.7347; 95% CI 0.5491 to 0.9203; p = 0.03460 respectively (Fig. 6).

#### Gene Ontology analysis of predicted targets of five up-regulated miRNAs

**Fig. 7:**
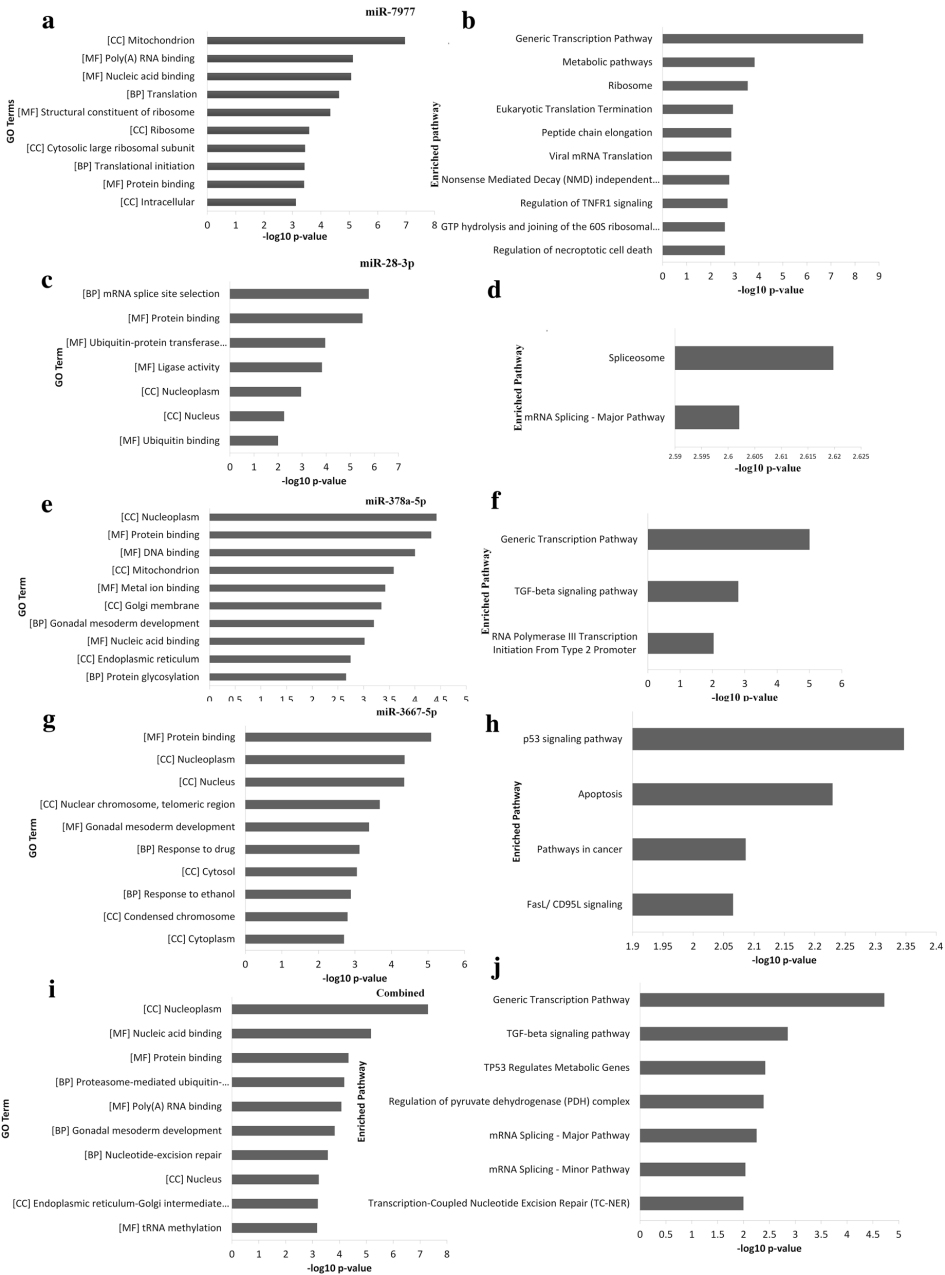
GO term analysis and enriched pathway analysis of the genes targetted by the 5 up-regulated miRNAs. Vertical axis depicts the GO terms and enriched pathways using KEGG and Reactome. **a**, **c**, **e**, **g**, **i** miR-7977, miR-28-3p, miR-378a-5p, miR-3667-5p combined GO term analysis. **b**, **d**, **f**, **h**, **j** miR-7977, miR-28-3p, miR-378a-5p, miR-3667-5p enriched pathway analysis

In order to study the role of these five up-regulated miRNAs in complicated *P. vivax* malaria the putative gene targets of these miRNAs were assessed using TargetScan with a cut off of 0.1 of total context score. A total of 3877, 107, 2307, 396 and 1960 target genes were predicted for hsa-miR-7977, hsa-miR-28-3p, hsa-miR-378-5p, hsa-miR-194-5p and hsa-miR-3667-5p respectively. Then the first 3000 targets in case of hsa-miR-7977 and all gene targets of rest of the miRNAs were then subjected to gene ontology (GO) analysis in DAVID v6.7 tool. At cutoff standard of p < 0.01, a total of 30, 7, 22 and 23 GO terms of biological process, molecular function and cellular component for hsa-miR-7977, hsa-miR-28-3p, hsa-miR-378-5p and hsa-miR-3667-5p was found for the complicated *Plasmodium vivax* group. No GO term was found for hsa-miR-194-5p using DAVID. A total of 30 GO terms were obtained for combined gene targets, which were common in any of the two miRNAs (Fig. 7a, c, e, g, i).

#### KEGG and reactome pathway analysis of the predicted targets of the up-regulated microRNAs

To gain an overall knowledge of these miRNA in complicated *P. vivax* infections, KEGG and Reactome pathway analysis was performed in DAVID v 6.7. The pathways were ranked significant as per their p value (p < 0.01). A total of 18, 2, 3, 4 and 7 pathways were significantly enriched by targeted genes of hsa-miR-7977, hsa-miR-28-3p, hsa-miR-378-5p and hsa-miR-3667-5p and for combined target genes of all five miRNAs. The top pathways in the combined analysis shown here are to be the ones involved in generic transcription pathway, TGF-beta signalling pathway and mRNA-splicing pathways Fig. 7b, d, f, h, j.

#### Protein-protein interaction (PPI) analysis

**Fig. 8:**
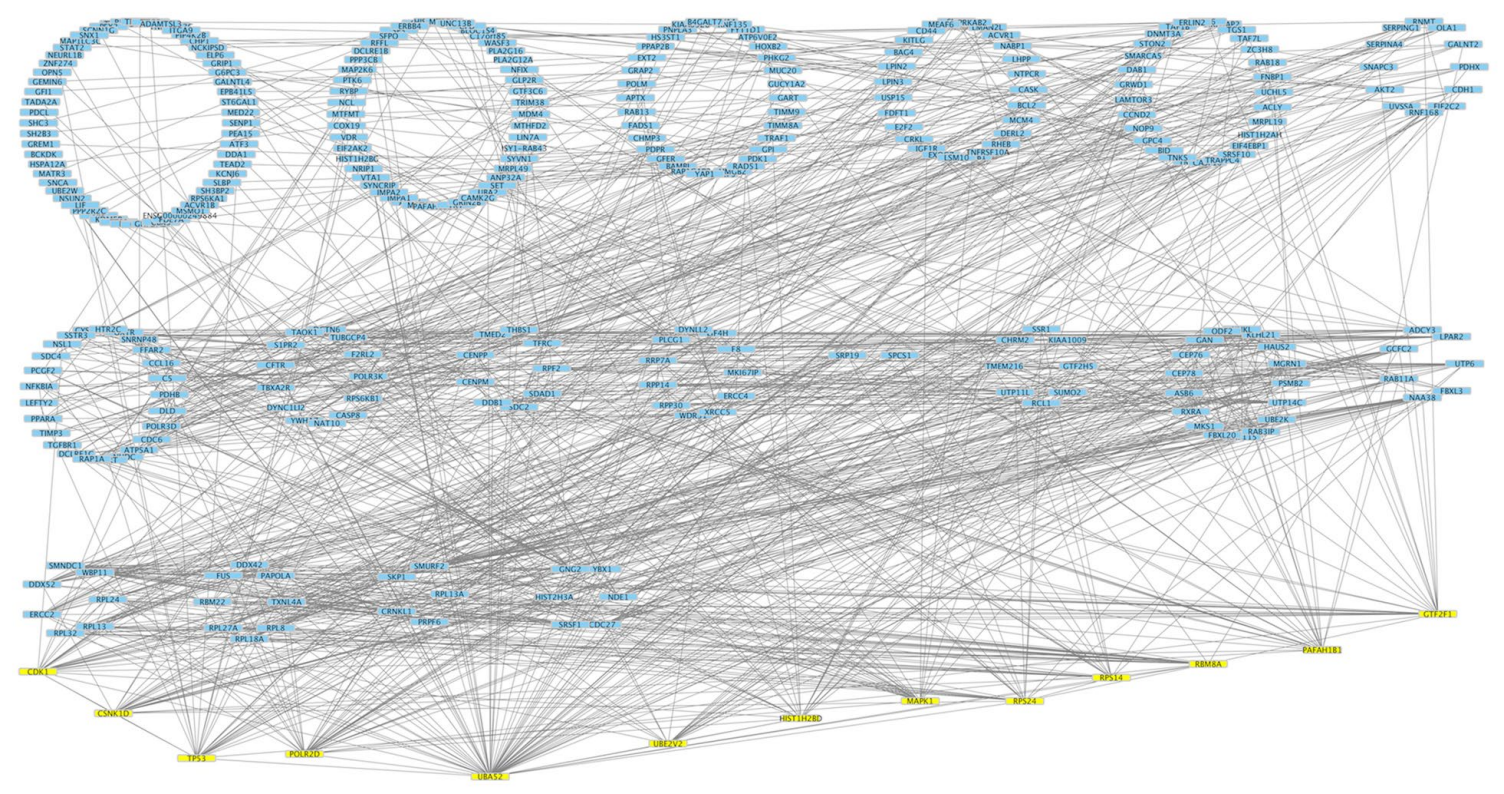
Protein-protein interaction network of the miRNA targets. Nodes represent targets and edges represent the interaction between the target genes. The nodes with STRING combined score of 0.9 were selected for network construction

**Fig. 9:**
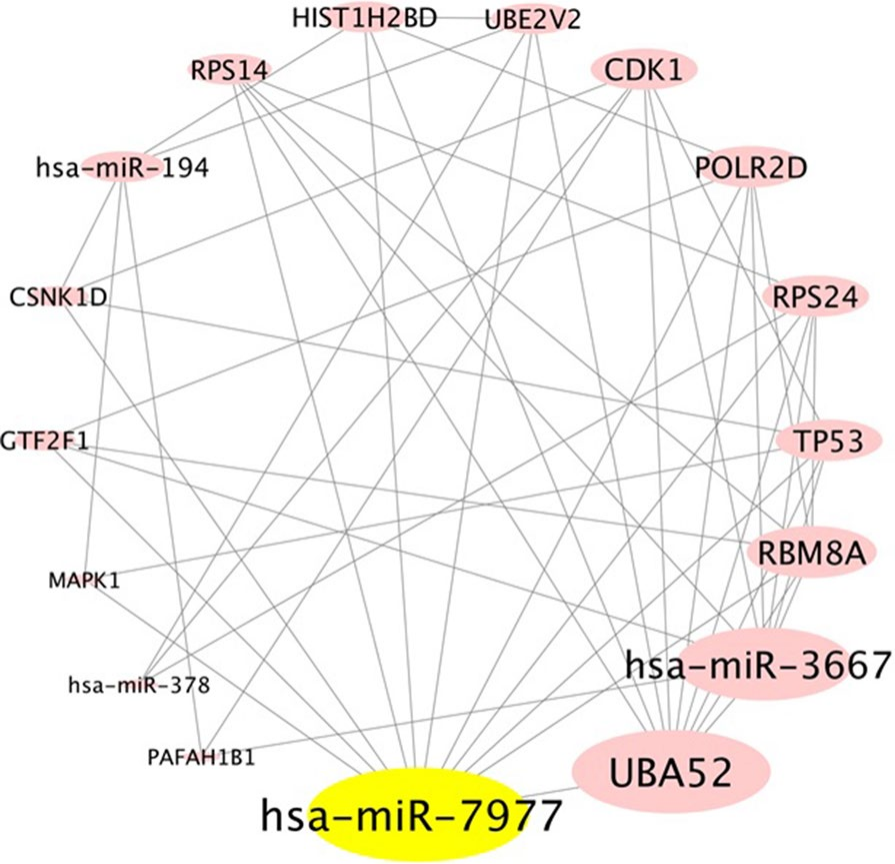
Relationship between mRNA–miRNA targets. The mRNAs with degree of more than 21 were selected for network construction. Nodes represent the highly interacting mRNA–miRNA network and edges represent the interaction between them

The summarized layout for the detailed analysis of PPI networks are shown in Additional file 2: Figure S1. The common gene targets between any of the two miRNAs were selected for the network construction using STRING version: 10.5 (Additional file 3: Figure S2). A total of 1624 nodes with 6445 edges were found in the network with a p value of 0.000398. The data obtained from STRING was uploaded into the cytoscape (Fig. 8). The nodes with combined score of 0.9 were then used to construct the network. The highest degree of 71 was found for UBA52. To construct mRNA–miRNA network the nodes with degree of more than 21 were selected. Out of 344 nodes, 13 nodes (UBA52, POLR2D, CSNK1D, TP53, CDK1, GTF2F1, PAFAH1B1, RBM8A, RPS24, RPS14, HIST1H2BD, MAPK1, UBE2V2) were found to have degree range from 21 to 71. The mRNA–miRNA network was constructed using cytoscape and hsa-miR-7977 was found to have the highest degree of 11 as compared to other miRNAs (Fig. 9). Hence, miR-7977 might be a major regulator of the complications and could be used as a potential biomarker for the prognosis of complications in *P. vivax* malaria.

## Discussion

In the recent years, the importance of severe *P. vivax* is being outweighed by *P. falciparum* which is known to be associated with fatal infections [36]. A number of studies from different geographical regions viz. Thailand, Brazil, Indonesia, Papua New Guinea and India have reported an increase in the number of severe malaria caused by *P. vivax* malaria [4, 37]. In *P. vivax* the mechanism initiating the transformation of uncomplicated malaria to severe *P. vivax* malaria still remains poorly understood. Increased deformability of red blood cells and almost absence of parasite sequestration in contrast to *P. falciparum* is capable of causing fatal manifestations [38]. An early detection with initiation of correct treatment and close monitoring of the patient would be lifesaving. In the previous studies, the association of various plasma detectable markers (PCT, CRP, ICAM, ANG2, TNFα and IP-10) with mortality in patients with severe or cerebral malaria have been reported [39]. Recently the research has focused mainly on the potential role of miRNA in the underlying normal and pathophysiological conditions of various diseases [20].

Till date only a handful of available studies have highlighted the probable link of miRNA in the pathogenesis and as a diagnostic marker of *P. falciparum* and *P. vivax* malaria. In a case study of acute *P. vivax* infection, Baro et al. [40] have reported a distinct series of differentially expressed miRNA associated with erythropoiesis. Another study has reported the levels of miR-451 and miR-16 to be significantly down regulated in the *P. vivax* infections as compared to normal subjects suggesting to be the probable biomarker of *P. vivax* malaria infections [41]. LaMonte et al. [42] have reported the impaired growth of parasites due to mRNA translation inhibition, which was observed due to increased accumulation of miR-451 and miR-223 in both the HbAS and HbSS erythrocytes. Study on *P. chabaudi* infected C57BL/6 mice has suggested the association of reprogramming of 19 distinct miRNA in female mouse liver with the development of protective immunity against blood stages of *P. chabaudi* infection [43]. Assaad et al. [44] have showed a significant change in the expression of let-7i, miR-150, miR-27a in the brain tissue of PbA infected WT mice, suggestive of their critical involvement in the triggering of the neurological syndrome. A recent study for the first time depicted the innate ability of normal RBCs to prevent infection by *P. falciparum* parasite by the transfer of the hAgo2-miRNA complexes via microparticles (MPs) to infected RBCs and thus down regulating of the expression of PfEMP1 [45]. Wang et al. have also described the potential role of miR-155 in host response to malaria via BBB dysfunction through microvascular leak and regulation of endothelial activation in cerebral malaria (CM) [46]. Another study conducted on controlled human blood-stage infection (CHMI) has shown the dichotomous miRNA expression distinguishing high-miR responders from low-miR responders with significantly enhanced antimalarial antibody responses in the high-miR responders group [47]. At present there is no approved diagnostic modality available to detect the severity of the *P. vivax* malaria [48]. In the present study we identified the potential whole blood miRNA marker for the probable diagnosis of complicated *P. vivax* malaria using miRNA microarray.

The present study is first of its type where miRNA microarray was carried on whole blood samples of *P. vivax* complicated and uncomplicated malaria patients in order to identify putative biomarker of complicated *P. vivax* malaria. A total of five miRNAs (hsa-miR-7977, hsa-miR-28-3p, hsa-miR-378-5p, hsa-miR-194-5p, hsa-miR-3667-5p) were found to be statistically significantly (p < 0.03) up-regulated in the complicated *P. vivax* group of patients as compared to the uncomplicated group of patients using qRT-PCR. The diagnostic potential for all these miRNAs were also assessed and was found to be significant with good sensitivity and specificity. Among these five miRNAs, hsa-miR-7977 had the highest degree of interaction in the mRNA–miRNA networks and thus might have a role to play in the pathogenesis of complicated *P. vivax* malaria. Previous studies have reported the validity of the reference genes being affected by the type of sample and experimental procedures used [49]. Therefore, in this study we have confirmed our selection of reference genes (U6 and U48) using four different softwares and thus used them for further q RT-PCR.

The information on the pathogenesis of severe anaemia in *P. vivax* remains majorly obscure. Molecular mechanisms underlying the inhibition of the erythroid cell proliferation and differentiation by *P. vivax* infection is complex and remains unclear [50]. Studies have provided the evidence of the dyserythropoiesis and ineffective erythropoiesis in *P. vivax* malaria patient’s bone marrow [51]. Ineffective erythropoiesis due to the *P. vivax* inhibited erythroid development has been reported to play a potential role in severe anaemia caused due to *P. vivax*  [52]. Recently the role of miR-7977 in the hematopoietic dysfunction of mesenchymal stromal cells by poly (rC) binding protein 1supression has been explained by Horiguchi et al. [53]. The significant upregulation of miR-7977 in the complicated group of patients obtained in this study may suggest their putative role in the regulation of erythropoiesis. However, further studies will be required to establish their probable function in complicated *P. vivax* malaria.

The TGF-β signalling pathway was found to be the most significantly regulated pathway in the present study. Depending on the environment and concentration, the TGF-β is capable of exhibiting both pro- and anti-inflammatory properties [54]. TGF-β is known to supress production of TNF-α and nitric oxide from macrophages and of IFN-γ and TNF-α from NK cells [55]. The simultaneous increase of TNF and IFN-γ in case of *P. vivax* malaria infections has been linked with severe disease progression [56]. Study by Omer et al. have reported the low circulating levels of TGF-β, and bioactive TGF-β produced by splenocytes in deadly infections with *Plasmodium berghei* and significant production of TGF- β in *P. chabaudi* or *P. yoelii* resolving infections [57]. Study by Perkins et al. [58] have also demonstrated the association of low levels of TGF- β1 and IL-12 with the malaria disease severity. In our study the TGF- β signalling pathways being the most effected pathway might have some role to play with the severe *P. vivax* infections.

In-silico analysis was performed for the five up-regulated miRNAs to identify possible role of these miRNAs in complicated *P. vivax* malaria. The targeted genes of up-regulated miRNA were found to be mainly enriched in the protein binding, nucleic acid binding, proteasome-mediated ubiquitin-dependent protein catabolic process, Poly (A) RNA binding, generic transcription pathway, TGF-beta signalling pathway, regulation of pyruvate dehydrogenase (PDH) and mRNA-splicing pathways. Generic transcription and m-RNA splicing pathways are one of the majorly regulated pathways by almost all the miRNAs in the present study. Our results are in accordance with the previous belief of the possibility, that *Plasmodium* might utilize the host miRNAs to regulate their gene expression [42, 59, 60]. In the present study, UBA52 was found to have the highest degree of interaction in the PPI network. Among the nuclear and cytoplasmic proteins, UBA52 has a role in the targeted cellular protein degradation, regulation of gene expression and also involved in the stress response. UBA52 is an important ubiquitin supplier of the ubiquitin pool and also is an important regulator of ribosomal-protein complex [61] clearly depicting the role in ubiquitin-dependent translation mechanism. The higher degree of interaction of this protein in complicated *P. vivax* group suggests its potential role in the gene regulation mechanism in the severe disease. However, future studies are required to evaluate the functions of the highly connected nodes in the pathogenesis of complicated *Plasmodium vivax* malaria.

## Conclusion

To summarize the present study, we found five miRNA which are up-regulated in the *P. vivax* complicated group of patients as compared to the uncomplicated group of patients. These miRNAs have a good sensitivity and specificity to be used as a predictor of severity in *P. vivax* malaria. hsa-miR-7977 was found to be highly up-regulated in complicated *P. vivax* and may have a role in exacerbating the disease pathology through UBA52 or TGF-beta signalling pathway. Hence, hsa-miR-7977 should be explored as a potential biomarker for differentiating complicated vs uncomplicated *P. vivax*. However, the findings of the present study need to be further explored in future studies to understand the disease pathogenesis.

## Additional files

**Additional file 1: Table S1.** Differentially expressed miRNAs in 1. Complicated vs uncomplicated P. vivax 2. Complicated P. vivax vs Healthy 3. Complicated P. vivax vs P. falciparum with p-value < 0.05.

**Additional file 2: Figure S1.** Summarized layout for the detailed analysis of PPI networks.

**Additional file 3: Figure S2.** Venn diagram analysis showing criteria used for the selection of common targets between any of the two miRNAs (http://www.interactivenn.net/).

## Abbreviations

AUC: area under curve
PPI: protein–protein interaction
ARDS: acute respiratory distress syndrome
AKI: acute kidney injury
IQR: interquartile range
PCT: procalcitonin
CRP: C-reactive protein
ICAM: intercellular adhesion molecule
ANG2: angiogenin
TNFα: tumor necrosis factor
IP-10: IFN-gamma-inducible protein10
ROC: receiver operating characteristic
GO: gene ontology
DAVID: Database for Annotation, Visualization and Integrated Discovery
KEGG: Kyoto Encyclopedia of Genes and Genomes
TGF: transforming growth factor
STRING: search tool for the retrieval of interacting genes/proteins
EC: endogenous control
UBA52: Ubiquitin A-52 Residue Ribosomal Protein Fusion Product 1
POLR2D: RNA polymerase II subunit D
CSNK1D: casein kinase 1 Delta
TP53: tumor protein P53
CDK1: cyclin-dependent kinase 1
GTF2F1: general transcription factor IIF subunit 1
PAFAH1B1: platelet activating factor acetylhydrolase 1b regulatory subunit 1
RBM8A: RNA binding motif protein 8A
RPS24: ribosomal protein S24
RPS14: ribosomal protein S14
HIST1H2BD: histone cluster 1 H2B family member D
MAPK1: mitogen-activated protein kinase 1
UBE2V2: ubiquitin conjugating enzyme E2 V2
