# Supplementary figures and images for: Screening and identification of potential novel biomarker for diagnosis of complicated Plasmodium vivax malaria
Source: J Transl Med. 2018 Oct 4;16:272. doi: 10.1186/s12967-018-1646-9 (PMC6172720; doi:10.1186/s12967-018-1646-9)

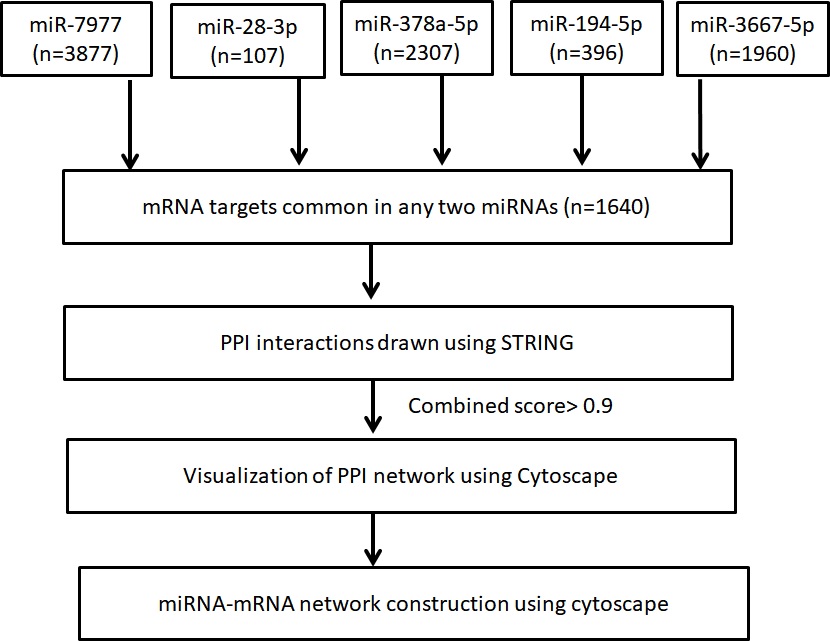

Supplement: Supplementary file 2 — Additional file 2: Figure S1. Summarized layout for the detailed analysis of PPI networks. [file 12967_2018_1646_MOESM2_ESM.jpg]

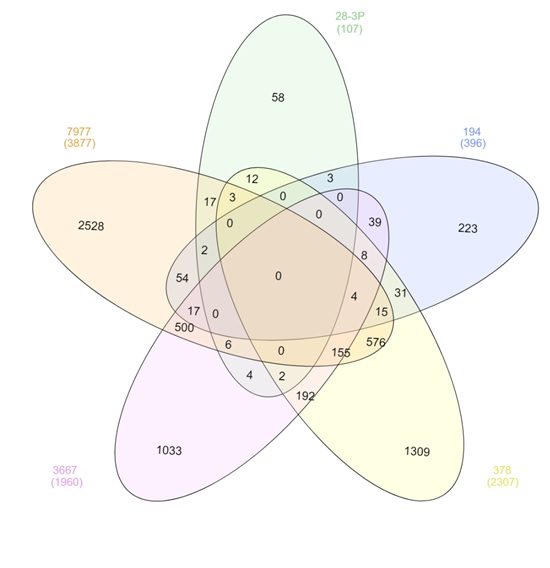

Supplement: Supplementary file 3 — Additional file 3: Figure S2. Venn diagram analysis showing criteria used for the selection of common targets between any of the two miRNAs (http://www.interactivenn.net/). [file 12967_2018_1646_MOESM3_ESM.jpg]
